# The Effect of Delivery Mode, ABO Blood Type, and Passive Smoking on Postpartum Depression: A Cross-Sectional Study in Saudi Arabia

**DOI:** 10.7759/cureus.38466

**Published:** 2023-05-02

**Authors:** Maisam H Alhammadi, Alwa I Almontashri, Ebtesam M Radwan, Maryam A Khouj, Afnan A Alsaif, Zainab A Alkhalifah, Manar K Alzahrani, Alaa A Basuliman, Wid Kattan, Nedaa M Bahkali

**Affiliations:** 1 Department of Medicine, King Abdulaziz University Faculty of Medicine, Jeddah, SAU; 2 Department of Obstetrics and Gynecology, King Abdulaziz University Faculty of Medicine, Jeddah, SAU

**Keywords:** abo blood group, passive smoking, type of delivery, major depression, postpartum blue, postpartum depression

## Abstract

Background

Postpartum depression (PPD) is a form of depression that can occur after childbirth and is characterized by feelings of sadness. It is a common psychological problem that affects women and children. This study aimed to assess the association between PPD and risk factors, such as delivery mode, ABO blood group, and passive smoking in Saudi Arabia.

Methods

PPD was assessed in this cross-sectional using an Arabic version of the Edinburgh postnatal depression scale through an online questionnaire distributed to women in Saudi Arabia between January and March 2022. The data were analyzed using SPSS version 26 (IBM Corp., Armonk, NY).

Results

A total of 354 postpartum women completed the questionnaire within six weeks of giving birth. Their mean age and BMI were 30.1±6.78 years and 25.98±5.84 kg/m^2^, respectively. PPD occurred in 56.2% of the participants. Elective cesarean section and operative vaginal delivery were associated with the presence of PPD symptoms in 17.6% and 7% of the women, respectively. The majority of those with third and fourth degrees and those who had instrumental assisted delivery had postpartum depression and this was statistically significant (p=0.017). About 26.6% of the participants were exposed to passive smoking, and 21.9% of them developed PPD. However, it was not statistically significant. Moreover, women with PPD were more likely to have blood type O+, followed by A+. Demographic factors did not show a significant correlation with developing PPD except for age (p=0.01), those who developed PPD were much younger on average than those who did not develop PPD (29.28±6.61 years vs. 31.15±6.86 years).

Conclusion

A significant association was found between PPD and the type of delivery. The association between PPD and passive smoking, ABO blood groups was insignificant. However, women who developed PPD were younger on average than those who did not develop PPD.

## Introduction

Depression is one of the most serious public health problems worldwide that can lead to disability [1]. According to the World Health Organization, approximately 10% of pregnant women and 13% of women who have given birth suffer from a mental disorder, particularly depression [2]. Postpartum depression (PPD) is an excessive feeling of sorrow and anxiety that often occurs within one to three weeks following birth [3]. This condition may begin at any time during the first year after childbirth and last for a few years [4,5].

PPD may limit a mother’s ability to provide quality care for her child and can cause difficulty with simple daily tasks, which decreases the mother’s quality of life and increase the child’s risk of developing long-term behavioral and emotional impairment [6]. It is associated with breastfeeding difficulties or early cessation of breastfeeding [7]. Moreover, a previous study showed that 11.74% of patients with PPD have suicidal ideation [8]. Psychological factors, such as recent traumatic life experiences, previous history of depression, and depression or anxiety during pregnancy, as well as social and lifestyle factors, such as lack of support from the partner and history of abuse, have been associated with PPD development [3].

Mode of delivery has been investigated as a risk factor for PPD. A study of 482 women in Brazil found no association between delivery mode and maternal depression [9]. By contrast, another study performed in Poland reported that women who had a cesarean section (CS) present more early signs of depression, particularly those who had an emergency CS, and experience more discomfort on discharge than those who had a vaginal delivery [10]. In the Arab region, few studies have explored the association between PPD and mode of delivery. A study conducted in Egypt concluded that women who underwent emergency CS had the highest prevalence of PPD (25%), followed by those who had elective CS (21%) and vaginal delivery (7%) [11]. In Riyadh, Saudi Arabia, a study evaluated the prevalence of PPD and its associated predictors, including unsupportive spouses, stressful life events, and cesarean delivery [12]. However, the association between the development of the condition and the different modes of delivery was not investigated.

The ABO blood group system is characterized by the expression of ABH antigens on the red blood cell surface [13]. A few studies reported an association between different ABO blood group types and various psychiatric disorders, such as depression, manic-depressive psychosis, and schizophrenia [14]. A systematic review of 27 cohort studies in 2021 found no association between ABO blood types and major depressive disorder [15]. By contrast, research conducted in the USA reported that individuals with blood type O have a higher risk of developing PPD [16].

Smoking, which is becoming more prevalent in Saudi Arabia, is another risk factor associated with PPD [17,18]. Several studies have explored the association between passive smoking and PPD development [19]. Furthermore, pregnant women exposed to passive smoking have a greater risk of premature labor, increased perinatal death, and reduced fetal development [20-23]. Other studies reported an association between passive smoking and maternal outcomes, such as PPD. A study conducted in the USA found that women who were exposed to passive smoking during pregnancy have twice the odds of developing depressive symptoms than those who were unexposed [24].

The existing literature suggests that the prevalence of PPD is higher in Saudi Arabian women. However, the low sample size, collection of data from a single location, and paucity of studies that compare PPD prevalence with its risk factors in the Saudi Arabian context warrant the motivation for further research. Thus, this cross-sectional study aimed to evaluate PPD and its associated risk factors in women in Saudi Arabia.

## Materials and methods

Study design and setting

This cross-sectional study was conducted using an online survey distributed through social media to collect data from January to March 2022.

Study participants and sample size

Participants included in this study were women who had been pregnant and given birth within the last six weeks in Saudi Arabia. Women who refused to participate and those who were previously diagnosed with mental illness were excluded. For this study, the estimated sample size was calculated with Raosoft sample size calculator [25]. The sample size was calculated based on a response rate of 50%, a confidence interval of 90%, and a margin of error of 5%, with an annual average of approximately 590,963 deliveries in the Kingdom of Saudi Arabia according to the most updated statistical publication from the General Authority for Statistics [26]. The largest required sample size was calculated to be 271. Data collectors from across the regions of the country were assigned to reach the sample from all across Saudi Arabia. Snowball sampling method was used in which the survey was sent to women who have recently given birth and they were therefore encouraged to also spread the questionnaire. To ensure that all women who were reached were filling out the questionnaire within six weeks of giving birth, a section of the questionnaire inquiring about the time since giving birth was included. 

Data collection tool

A pre-designed questionnaire was prepared to collect data on participants’ demographic characteristics, smoking status during pregnancy, chronic diseases, passive smoking exposure, ABO blood group type, mode of delivery, and other risk factors.

The Edinburgh Postnatal Depression Scale (EPDS) was used to assess PPD. This scale helps health professionals identify mothers who are suffering from PPD, a debilitating disease that lasts longer than the “baby blues,” which may occur in the first week after delivery. The EPDS contains 10 items. For each item, mothers are required to select one of four options that best describe their feelings during the previous week. Most mothers complete the scale in less than 5 min. Responses are given a score of 0, 1, 2, or 3 based on the severity of the ailment. Items 3-10 are reverse scored (i.e., 3, 2, 1, and 0). The final score is calculated by adding the scores for all 10 items [27]. The most widely used cut-off values are 10 for moderate depression and 13 for serious depression. The same cut-off values are used to distinguish between “possible” and “likely” depression diagnoses [28,29].

Participants had the choice either to fill out the questionnaire in Arabic or English. Regarding the EPDS scale, the validated English and Arabic versions were used. Regarding additional questions such as smoking exposure data, they were constructed using the aid of similar literature and included items inquiring about exposure to smoke during pregnancy. The first section was about active smoking, its type, and whether they quit smoking during pregnancy. On the other hand, the other section inquired more about passive smoking, including the number of days of exposure to smoke at home. The questions were revised with OBGYN consultants and translated by two different authors into Arabic; while two other different authors translated them from Arabic back into English in order to ensure correct interpretation.

Statistical analysis

Microsoft Excel 2016 and SPSS version 26 (IBM Corp., Armonk, NY) were used for data entry and analysis, respectively. The chi-square test (χ2) was performed to assess the association between variables, and qualitative data are expressed as numbers and percentages. The Mann-Whitney U test was conducted to compare non-parametric variables, and quantitative data are expressed as means and standard deviations (mean±SD). Statistical significance was set at a p-value of 0.05.

Research ethics 

This study was approved by the biomedical ethics committee of King Abdulaziz University Hospital (reference no. 22-22). All study participants were informed of the research objectives, and informed consent was obtained from them at the start of the questionnaire.

## Results

A total of 354 women completed the questionnaire within six weeks postpartum. The mean age and body mass index of the participants were 30.1±6.78 years and 25.98±5.84 kg/m^2^, respectively. About 48% were housewives, and the remainder were either employed (35.9%) or students (16.1%). Only 16.1% had chronic diseases, and the most common diseases were asthma (29.8%), hypertension (21%), diabetes mellitus (8.7%), and thyroid disorders (8.7%). Table 1 shows the rest of the sociodemographic data of the participants.

 

**Table 1:** Distribution of studied participants according to their demographic characteristics, smoking during pregnancy, and chronic diseases (N=354).

Variable	
Quantitative	Mean ±SD
Age	30.1 ±6.78
BMI	25.98 ±5.84
Qualitative	N (%)
Educational level	
Bachelor’s degree	247 (69.8)
High school diploma	2 (0.6)
Less than high school diploma	7 (2)
Master degree	23 (6.5)
PhD degree	9 (2.5)
Secondary school	66 (18.6)
Place of residence	
Central region	60 (16.9)
Eastern region	115 (32.5)
Northern region	32 (9)
Southern region	54 (15.3)
Western region	93 (26.3)
Employment status	
Employee	127 (35.9)
Housewife	170 (48)
Student	57 (16.1)
Family monthly income	
<3000	24 (6.8)
>8000	173 (48.9)
3000-5000	57 (16.1)
5000-8000	100 (28.2)
Did you smoke during your pregnancy?	
Yes	14 (4)
No	340 (96)
Do you have any chronic diseases?	
Yes	57 (16.1)
No	297 (83.9)

 

**Figure 1:**
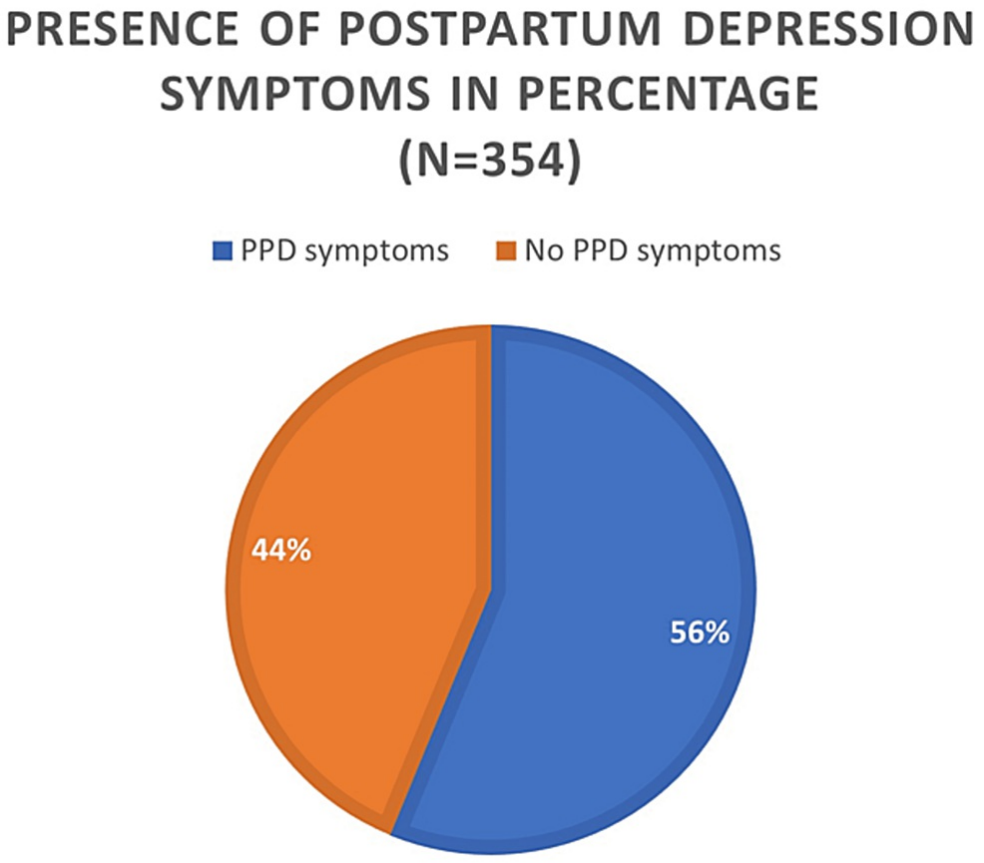
Percentage distribution of the women according to the presence of postpartum depression symptoms.

**Figure 2:**
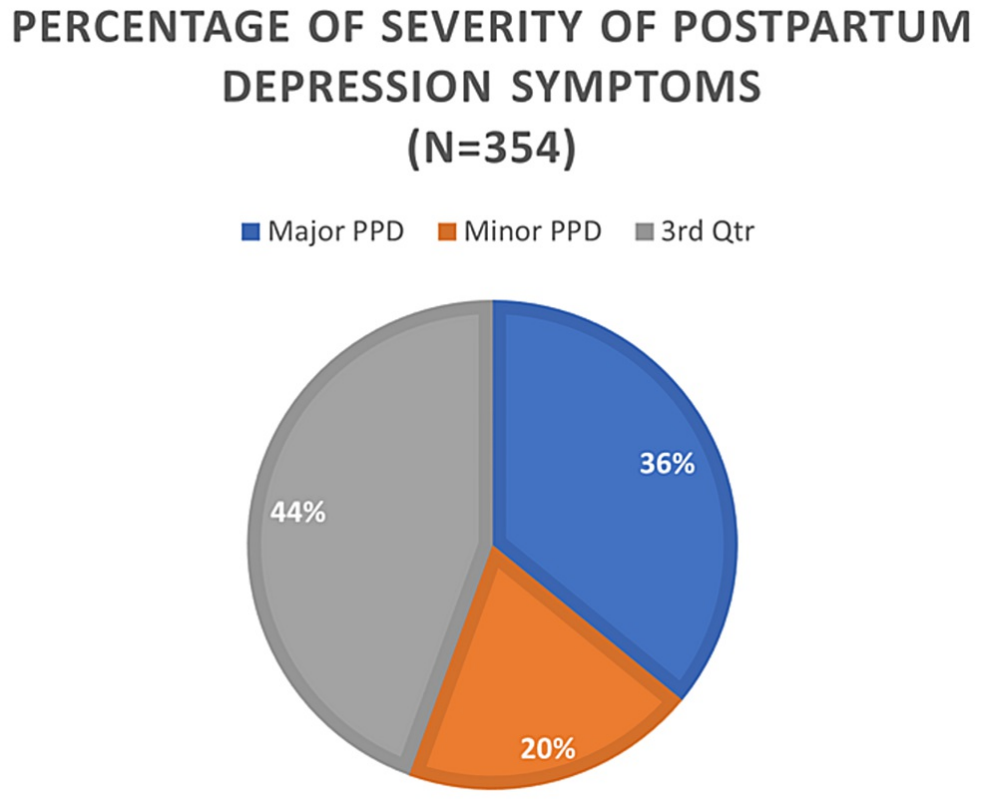
Percentage distribution of the women according to the severity of postpartum depression symptoms.

PPD occurred in 56.2% of the participants (Figure 1), with 19.8% and 36.4% having moderate and major PPD, respectively (Figure 2). Participants who developed PPD were significantly younger on average than those who did not develop PPD (29.28± 6.61years vs. 31.15± 6.86 years) with p-value =0.01, but other demographic variables were not significantly associated with the development of PPD (Table 2).

 

 

 

 

 

 

 

**Table 2:** Relationship between PPD and participants’ demographic characters and chronic diseases (N=354). N.B.: * = Mann-Whitney U test, PHD = Doctor of Philosophy, BMI = Body Mass Index

Variable	Postpartum depression (PPD)		χ2		P-value
	No	Yes			
Qualitative	N (%)	N (%)			
Educational level					
Bachelor’s degree	102 (65.8)	145 (72.9)	6.42		0.267
High school diploma	2 (1.3)	0 (0.0)			
Less than high school diploma	4 (2.6)	3 (1.5)			
Master degree	14 (9)	9 (4.5)			
PHD degree	4 (2.6)	5 (2.5)			
Secondary school	29 (18.7)	37 (18.6)			
Place of residence					
Central region	28 (18.1)	32 (16.1)	3.04		0.551
Eastern region	45 (29)	70 (35.2)			
Northern region	16 (10.3)	16 (8)			
Southern region	21 (13.5)	33 (16.6)			
Western region	45 (29)	48 (24.1)			
Employment status					
Employee	55 (35.5)	72 (36.2)	3.57		0.168
Housewife	81 (52.3)	89 (44.7)			
Student	19 (12.3)	38 (19.1)			
Family monthly income					
<3,000	11 (7.1)	13 (6.5)	1.4		0.704
>8,000	79 (51)	94 (47.2)			
3,000-5,000	21 (13.5)	36 (18.1)			
5,000-8,000	44 (28.4)	56 (28.1)			
Do you have any chronic diseases?					
Yes	28 (18.1)	29 (14.6)		0.78	0.375
No	127 (81.9)	170 (85.4)			
Quantitative	Mean ±SD	Mean ±SD			
Age	31.15± 6.86	29.28± 6.61	2.58*		0.01
BMI kg/m^2^	25.9 ± 4.91	26.05 ± 6.48	1.8		0.855

 

In this study, 52.1% of the participants were passive smokers. The number of days of exposure to passive smoking at home did not significantly affect the probability of developing PPD as presented in Table 3.

 

**Table 3:** Association between PPD and the number of days per week of cigarette smoke exposure at home during pregnancy and being an ex-smoker (N=354).

Variable	Postpartum depression (PPD)		χ2	P-value
	Yes No. (%)	No No. (%)		
Were you a passive smoker during your pregnancy? (you inhale cigarette smoke from smokers around you)				
No	121 (78.1)	139 (69.8)	3.01	0.082
Yes	34 (21.9)	60 (30.2)		
During your pregnancy, how many days in a week did you smell cigarette smoke at home?				
0 days in a week	119 (76.8)	139 (69.8)	2.13	0.343
1-4 days in a week	21 (13.5)	36 (18.1)		
5-7 days in a week	15 (9.7)	24 (12.1)		
Are you an ex-smoker?				
No	146 (94.2)	187 (94)	0.008	0.93
Yes	9 (5.8)	12 (6)		
If you were a smoker, when did you stop smoking in your last pregnancy? (before how many weeks /months/years) (No.:21)				
Quit before pregnancy	3 (1.9)	7 (3.5)	4.24	0.374
Quit at 1st 3 months	6 (3.9)	3 (1.5)		
Quit at 2nd 3 months	0 (0.0)	1 (0.5)		
Quit at last 3 months	0 (0.0)	1 (0.5)		

 

**Figure 3:**
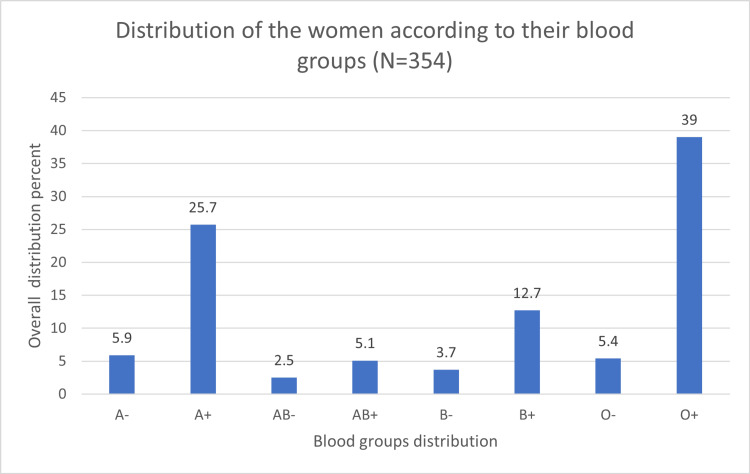
Distribution of the women according to their blood groups.

**Figure 4:**
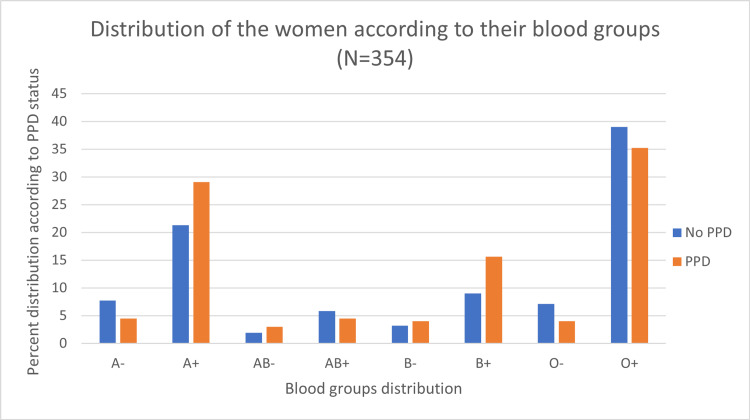
Association between blood type groups (eight groups) and PPD (N=354).

Figure 3 shows the distribution of blood groups; the most common blood group was O+ (39%), followed by A+ (25.7%). Women with blood type O showed the highest PPD rate (43.9%). However, these findings were not statistically significant (p=0.157; Figure 4).

 

 

 

 

 

With regard to the obstetric and delivery data, 71.4% have less than four previous deliveries, only 9.3% have more than five previous deliveries. The mean duration since the last delivery was 3.68±1.76 weeks. More than half (50.6%) had vaginal delivery without any problems within six weeks postpartum. A significant association was noted between the occurrence of PPD and participants who had any complications during pregnancy (p<0.05). 26.3% of the participants had complications during pregnancy, and the most common complications were gestational diabetes mellitus (40.8%) and placenta-related complications (27.9%). Other pregnancy related complications were shown in Table 4.

 

**Table 4:** Association between PPD and participants’ obstetric history. N.B.: * = Mann-Whitney U test, ICU = Intensive care unit

Variable	Postpartum depression (PPD)		χ2	P-value
	No	Yes		
Qualitative	N (%)	N (%)		
Type of Delivery in the last birth				
Elective c-section	25 (16.1)	35 (17.6)	11.98	0.017
Emergency c-section	25 (16.1)	26 (13.1)		
Vaginal delivery with (ventose and forceps)	2 (1.3)	14 (7)		
Vaginal delivery with 3rd & 4th grade tears	7 (4.5)	20 (10.1)		
Vaginal delivery without any complications	96 (61.9)	104 (52.3)		
Did you have any complication during pregnancy?				
Yes	29 (18.7)	64 (32.2)	8.13	0.004
No	126 (81.3)	135 (67.8)		
The pregnancy was				
Single fetus	151 (97.4)	191 (96)	1.22	0.746
Twins	0 (0.0)	1 (0.5)		
Triplet	1 (0.6)	1 (0.5)		
Triplets or more	3 (1.9)	6 (3)		
Birth status				
Normal delivery (after 37 week)	140 (90.3)	171 (85.9)	1.57	0.209
Preterm delivery (before 37 week)	15 (9.7)	28 (14.1)		
Did you experience constant vomiting during pregnancy that lead to hospital admission?				
No	140 (90.3)	170 (85.4)	1.91	0.166
Yes	15 (9.7)	29 (14.6)		
Baby status after birth				
The baby needed to be admitted in to the (ICU)	13 (8.4)	28 (14.1)	2.74	0.097
The baby was in good health	142 (91.6)	171 (85.9)		
Baby gender				
Boy	71 (45.8)	90 (45.2)	0.01	0.913
Girl	84 (54.2)	109 (54.8)		
Use of anesthesia or analgesia?				
Yes	101 (65.2)	139 (69.8)	0.87	0.349
No	54 (34.8)	60 (30.2)		
Quantitative	Mean ±SD	Mean ±SD		
Length of hospital stay (in days)	2.42 ±1.83	3.52 ±6.15	0.85*	0.391
Baby birth weight in kilograms	2.88 ±0.78	2.71 ±0.74	1.24*	0.213

 

Statistically significant association was found between the mode of delivery and PPD occurrence (p=0.017). Of women who had PPD, 17.6% had elective CS and 7% had operative vaginal delivery. Moreover, the majority of those with 3rd and 4th degree and those who had instrumental assisted delivery has PPD, and this was statistically significant. By contrast, a non-significant association was found between PPD and all other obstetric history (p>0.05). The results are listed in Table 4.

 

Table 5 shows that participants who had stressful life events, who experienced intimate partner violence and a lifetime history of physical and/or sexual abuse, whose last pregnancy was unintended/unwanted pregnancy, who had negative attitudes toward pregnancy, who had fear of childbirth, and who had body image dissatisfaction had a significantly higher incidence of PPD (p<0.05). Meanwhile, those who had a history of premenstrual syndrome or premenstrual dysphoric disorders, anxiety symptoms and disorders, sleep disturbances, breastfeeding difficulty, childcare stress, or a family history of PPD in the first-degree relatives also had a significantly higher incidence of PPD (p<0.05).

 

**Table 5:** Association between PPD and participants’ exposure to stressful life events, past psychological experiences, and family history of PPD and psychological disorders (N=354).

Variable	Postpartum depression (PPD)		χ2	P-value
	No No. (%)	Yes No. (%)		
Do you have any stressful life events?				
No	139 (89.7)	132 (66.3)	26.45	<0.001
Yes	16 (10.3)	67 (33.7)		
Have you experienced intimate partner violence and lifetime history of physical and/or sexual abuse?				
No	150 (96.8)	184 (92.5)	3.03	0.081
Yes	5 (3.2)	15 (7.5)		
Is your last pregnancy unintended/unwanted pregnancy?				
No	128 (82.6)	144 (72.4)	5.11	0.024
Yes	27 (17.4)	55 (27.6)		
Did you have negative attitudes toward pregnancy?				
No	148 (95.5)	152 (76.4)	24.59	<0.001
Yes	7 (4.5)	47 (23.6)		
Did you have fear of childbirth?				
No	66 (42.6)	39 (19.6)	22.06	<0.001
Yes	89 (57.4)	160 (80.4)		
Do you have body image dissatisfaction?				
No	69 (61.9)	83 (41.7)	14.26	<0.001
Yes	59 (38.1)	116 (58.3)		
Do you have history of premenstrual syndrome or premenstrual dysphoric disorder? (symptoms: mood changes, headache, abdominal pain)				
No	72 (46.5)	58 (29.1)	11.23	0.001
Yes	83 (53.5)	141 (70.9)		
Do you have anxiety symptoms and disorders?				
No	131 (84.5)	99 (49.7)	46.27	<0.001
Yes	24 (15.5)	100 (50.3)		
Do you have sleep disturbance?				
No	108 (69.7)	80 (40.2)	30.4	<0.001
Yes	47 (30.3)	119 (59.8)		
Do you have breastfeeding difficulty?				
No	114 (73.5)	109 (54.8)	13.17	<0.001
Yes	41 (26.5)	90 (45.2)		
Do you have childcare stress (inconsolable infant crying, difficult infant temperament, or infant sleep disturbance)?				
No	115 (74.2)	85 (42.7)	35.13	<0.001
Yes	40 (25.8)	114 (57.3)		
Have you done any abdominal surgery?				
No	119 (76.8)	147 (73.9)	0.39	0.53
Yes	36 (23.2)	52 (26.1)		
Did you have miscarriages?				
No	109 (70.3)	131 (65.8)	0.8	0.369
Yes	46 (29.7)	68 (34.2)		
Do you have family history of postpartum depression? (1st degree relative)				
No	152 (98.1)	175 (87.9)	12.67	<0.001
Yes	3 (1.9)	24 (12.1)		
Do you have family history of any psychological disorder? (1st degree relative)				
No	145 (93.5)	162 (81.4)	11.15	0.001
Yes	10 (6.5)	37 (18.6)		

## Discussion

Postpartum depression

PPD has become an increasing cause of concern for women of various races, particularly Arabs. A study conducted in Al-Madina, Saudi Arabia, showed consistent results with our findings in which more than half of the participants suffered from PPD. An Egyptian study revealed that only one-third of their sample had PPD [30,31]. We found that approximately 50% of our group had PPD, with the majority having the major type, which was considerably greater than previous surveys performed in Western countries in 2015 (23.9%) and 2019 (20.9%) [29,32]. Considering our study was made during the COVID-19 era, this might have been a contributor to the heavy psychological distress of most people and may be responsible for this sharp increase. 

Among the demographic characteristics, only age was statistically significantly associated with PPD. Participants who developed PPD were younger than those who did not develop PPD, which is in line with the findings of 2021 Turkish and Indian studies [33,34]. This could be attributed to the lack of life experience and support at this age. By contrast, postpartum, women older than 25 years exhibited higher rates of depression (40%) than those who were younger (35.6%). in research performed in Riyadh, Saudi Arabia [12]. The disparity in results could be due to the different categorizations of the participants’ demographic data. A study among Jordanian women reported that age is not a reliable predictor of PPD [35]. The remaining demographics of our participants did not exhibit relevance, which is also supported by previous studies [33,36].

The majority of the participants were overweight, and almost half were housewives, which is comparable with other studies conducted among Saudi Arabian women [37]. The rest of the sociodemographic characteristics, such as educational level and income, exhibited no significant associations with the development of PPD symptoms. However, these findings contradict the Saudi Arabian research by Almarzouki et al., who found that all of the preceding sociodemographic characteristics were strongly associated with the occurrence of PPD symptoms [29]. This difference in results could be attributed to the inclusion of participants from all around the country in this paper, whereas the literature by Almarzouki et al. only included participants from the city of Jeddah; more accurate results could be obtained if postpartum women from each region of the country were separately studied.

PPD and smoking

The smoking status of the patients (including passive smoking) was not statistically significantly associated with PPD symptom development. A Chinese prospective cohort study found that women exposed to second-hand smoke had a significantly higher risk of perinatal depression and suicidal ideation [38]. This could be attributed to increased stress caused by passive smoking. Several studies found that the chemical nicotine present in cigarettes causes a physiological reaction that brings about stress [39,40]. Studies conducted in China and Thailand found that the majority of their participants were passive smokers, compared to only a quarter of our participants being exposed to second-hand smoke [41,42]. This could be attributed to extremely high smoking rates in China and Thailand and the difference in a number of smokers among the total sample size.

Mode of delivery and PPD

A statistically significant relationship between the mode of delivery and the occurrence of PPD was detected in this paper. This finding is supported by a previous study done in Argentina where C-section was detected as a significant risk factor of PPD independent of all other risk factors [43]. This is also consistent with findings from other studies where C-sections were also significantly associated with higher rates of PPD [44-46]. One study specifically discussed the relationship between emergency C-sections and found it to be a risk factor for developing PPD [47]. In contrast, other studies have claimed that there was no such association between mode of delivery and PPD [48-51]. Depression associated with cesarean birth may result from an extended hospital stay, increased intraoperative complication rates and infection risk, and greater medical care expenses [52,53].

ABO blood type and PPD

ABO although the majority of PPD patients in our study had blood type O+ followed by type A+, we did not find any significant differences in PPD occurrence between ABO blood groups. A previous Chinese study showed a significant association between blood group A and PPD and they concluded that blood type O carries the lowest risk [2,54] in contrast to our study where such differences cannot be concluded. This could be explained by differences in the population and ethnic makeup.

Partner abuse and PPD

PPD was considerably more prevalent among participants with a history of intimate partner abuse, stressful life events, unwanted pregnancy in the past, negative thoughts about getting pregnant, and body dissatisfaction than in those without these characteristics. Similar findings were obtained in a study by Alsayed et al., who found a statistically significant association between PPD and a history of prior depression, tough life experiences, and attitude toward pregnancy [32]. The physical strain of pregnancy and delivery, together with considerable hormonal changes throughout pregnancy, can induce and eventually lead to PPD. A woman’s overall mental health history and a history of traumatic or stressful experiences may also put her in a bad mood. Additionally, similar to the findings of AlGhamdi et al., women who experienced pregnancy problems and whose newborns required intensive care unit care had a greater incidence of PPD than those who did not [55]. This may be because women worry more about their children, which raises their stress levels and can make them more likely to experience PPD. By contrast, the presence of any maternal chronic illness did not affect PPD rates in this study, contrary to the study by AlGhamdi et al. [55].

Limitations and recommendations

The main limitation of this study was the sampling method; a random sample of women from all over Saudi Arabia was recruited via an online questionnaire. An ideal sample recruitment would be in obstetric clinics within the women’s six-week postpartum appointment to decrease the number of unconscientious responses and ensure that all women properly fit the inclusion criteria. However, this was not possible because of hospital and clinic restrictions implemented during the COVID-19 pandemic. Participant recall bias was another limitation, as a large part of the questionnaire is solely based on self-reported data due to the cross-sectional design of the study. Hospitals should adopt early psychiatric screening programs for PPD. A different study design, such as a prospective study, is expected to yield far more accurate data for future researchers on this subject. 

In addition, women with previously diagnosed mental illnesses, including depression, were excluded from the study to prevent previous psychological symptoms from affecting the interpretation of PPD symptoms. Including women with previously diagnosed psychological diseases in future studies may enable a comparison of the postpartum mental state in women who were previously diagnosed vs. undiagnosed and associate it with the mode of delivery.

## Conclusions

PPD was significantly higher in those who had undergone CS, those who had operative vagina deliveries, and those who sustained third- and fourth-degree tears, establishing a correlation between mode of delivery and PPD. However, the ABO blood group and passive smoking showed no significance with developing PPD as well as other demographic factors with the exception of age where those who were younger were at a significantly higher risk of PPD. Based on our findings we recommend that PPD screening should be implemented at postpartum visits, especially for those who are younger in age as they lack experience and practice.
